# From data to treatment plan: An AI‐driven path for automated breast radiotherapy planning

**DOI:** 10.1002/acm2.70491

**Published:** 2026-03-02

**Authors:** P. Gallego, E. Ambroa, J. Pérez‐Alija, J. C. Julià, N. Jornet, A. Matas, C. Anson, A. Mera, N. Tejedor, H. Vivancos, A. Ruiz, M. Barceló, A. Dominguez, V. Riu, J. Roda, P. Carrasco, S. Balocco, O. Díaz

**Affiliations:** ^1^ Department of Radiation Oncology‐Medical Physics Hospital de la Santa Creu i Sant Pau Barcelona Spain; ^2^ Department of Radiation Oncology Hospital de la Santa Creu i Sant Pau Barcelona Spain; ^3^ Servei de Física i Protecció Radiològica Hospital del Mar Barcelona Spain; ^4^ Department of Mathematics and Computer Science University of Barcelona Barcelona Spain; ^5^ Computer Vision Center Bellaterra Spain; ^6^ Institut de Recerca Sant Pau (IR SANT PAU) Barcelona Spain

**Keywords:** artificial intelligence, automated treatment planning, breast cancer, deep learning, radiotherapy

## Abstract

**Background:**

Breast cancer is one of the most prevalent malignancies in women, with radiotherapy (RT) playing a key role in its treatment. Advances in RT techniques, such as 3D conformal radiotherapy (3D‐CRT) and intensity‐modulated radiotherapy (IMRT), have improved dose precision and reduced side effects. However, RT modality selection and treatment planning remain manual, time‐consuming, and subject to variability.

**Purpose:**

This study presents and validates TARS‐B (Treatment Automation and Radiotherapy Selection for Breast Cancer), an automated framework that combines a deep learning‐based decision‐making module (DMF) for selecting the optimal RT technique and a fully automated treatment planning system (ATP) for generating deliverable plans that meet clinical quality standards and are deemed acceptable for clinical use.

**Materials and Methods:**

TARS‐B functions in two stages. First, the DMF analyzes individual patient data to determine whether 3D‐CRT or IMRT is more appropriate. Second, the ATP generates the corresponding treatment plan. For 3D‐CRT, a field‐in‐field (FiF) method is used to enhance dose homogeneity and minimize hotspots. For IMRT, the DMF provides neural network‐based dose predictions, which are used to generate constraints for organs‐at‐risk (OARs). Both processes are fully scripted within the treatment planning system (TPS).

The framework was tested on 60 breast cancer patients: 30 originally treated with 3D‐CRT and 30 with IMRT. Two analyses were conducted. First, the ATP's performance was evaluated by comparing automated plans with their manually generated clinical counterparts for both techniques. Second, the full TARS‐B pipeline was assessed by applying the DMF to select the RT modality and automatically generating the plan, comparing results to the original clinical plans. Dosimetric parameters, including planning target volume (PTV) coverage, OAR constraints, and low‐ and intermediate‐dose bath, were analyzed. Planning times were also compared.

**Results:**

No statistically significant differences (p>0.005) were found between manual and automated plans in key dosimetric metrics, including PTV coverage (V95%), hotspots (V105%), and OAR constraints, for both 3D‐CRT and IMRT. TARS‐B confirmed the appropriateness of 3D‐CRT in all patients originally treated with it and recommended re‐planning with 3D‐CRT for 15 of 30 IMRT cases. Of these, 14 re‐plans met all criteria; one failed due to anatomical anomalies.

Re‐planning led to a reduction in low‐dose bath (up to 2800 ${\mathrm{cm}}^{3}$) and intermediate‐dose bath (up to 3000 ${\mathrm{cm}}^{3}$). The reduction in intermediate‐dose bath was statistically significant (p<0.005). Planning times decreased substantially: from 157.4±116.2 to 2.0±1.3 min for IMRT, and from 112±70 to 5±4 min for 3D‐CRT (p<0.005).

**Conclusions:**

TARS‐B effectively automates both the selection of the most appropriate RT technique and the generation of high‐quality treatment plans. This framework improves workflow efficiency, reduces planning time, and preserves dosimetric quality, highlighting its potential for clinical implementation in breast cancer RT.

## 1 INTRODUCTION

Breast cancer remains one of the most common malignancies globally,^1^ and radiotherapy (RT) is a critical component of its management, commonly in the adjuvant setting following surgery. Over the past decades, advancements in RT techniques, including three‐dimensional conformal radiotherapy (3D‐CRT) and intensity or volumetric ‐modulated radiotherapy (IMRT or VMAT), have significantly improved the precision of dose delivery, allowing better sparing of surrounding healthy tissues and reducing long‐term side effects.^2^ While both IMRT and VMAT offer superior dose conformity and homogeneity, particularly in complex anatomical cases,^3^, ^4^ 3D‐CRT remains an effective treatment option for most patients.

In line with this, the *Choosing Wisely* campaign^5^ by the American Society for Radiation Oncology (ASTRO), launched in 2014, has issued recommendations to guide the appropriate use of advanced RT techniques. The campaign advises against the routine use of IMRT for whole‐breast radiotherapy, as similar clinical outcomes can be achieved with 3D‐CRT in the majority of cases. IMRT is recommended only for those patients who show more complex anatomies or clinical scenarios where superior tissue sparing is necessary.^6^ Since 3D‐CRT provides equivalent clinical results in most cases while requiring fewer resources, lower costs, and simpler treatment planning, it remains the preferred technique for whole‐breast radiotherapy in standard cases.

In parallel, recent advancements in artificial intelligence (AI) have driven notable progress in radiotherapy planning, with numerous studies focusing on dose prediction and treatment optimization.^7^, ^8^, ^9^, ^10^ Artificial neural networks have been extensively applied to predict three‐dimensional dose distributions, primarily as a research tool, but their integration into clinical workflows remains limited. While these models demonstrate potential for improving treatment planning, their predictions are not yet widely used in real‐world clinical decision‐making

In our previous work,^11^ we developed a decision‐making framework (DMF) based on neural networks capable of selecting the most appropriate RT technique for breast treatments, either 3D‐CRT or IMRT, by analyzing patient anatomy and delineated structures. This DMF not only streamlines the modality selection process but also ensures that IMRT is reserved for those cases where its benefits over 3D‐CRT are dosimetrically significant. Although VMAT has been reported to improve conformity and reduce treatment time compared to IMRT in breast cancer patients,^12^, ^13^, ^14^ it was not included in our framework because it is not routinely used in our institution for whole‐breast treatments, primarily due to concerns regarding the increased low‐dose bath to surrounding healthy tissues and the lack of clear clinical benefit over IMRT in standard cases.

In recent years, automation in treatment planning has gained traction, with many centers leveraging the scripting capabilities of commercial treatment planning systems to streamline workflows and improve consistency. Studies have demonstrated the use of scripting to automate plan generation, reducing manual intervention and enhancing efficiency.^15^, ^16^

Additionally, scripting frameworks have been applied to automate field‐in‐field (FiF) planning—an approach that uses subfields to improve dose uniformity—for 3D‐CRT breast cancer, improving homogeneity and reducing hotspots while minimizing variability across clinical settings.^17^

Despite significant advancements in RT techniques and the integration of AI into treatment workflow, a critical knowledge gap remains in developing fully automated workflows that not only predict the optimal RT technique but also generate complete, clinically acceptable treatment plans.

Existing studies primarily focus on isolated AI applications, such as dose prediction^9^, ^18^, ^19^, ^20^ or the automation of specific aspects of treatment planning, but they do not integrate this information into a fully automated treatment planning workflow. As a result, clinicians remain responsible for translating these predictions into deliverable plans, making key decisions about the treatment technique, or manually creating the plans based on AI‐generated outputs. While these approaches have demonstrated promising results, they still lack full automation, limiting their practical application in clinical workflows.

Since comprehensive frameworks that integrate AI‐driven decision‐making with fully automated plan generation across different RT modalities are still scarce, this study aims to bridge the gap between technological innovation and practical clinical application by providing an end‐to‐end automation framework for breast patients. In contrast to previous studies that focused either on automated planning or on decision‐support, the present work introduces a fully integrated end‐to‐end framework that combines both components.

Building on this foundation, the aim of this study is to develop and clinically validate this end‐to‐end automated treatment framework (TARS‐B, Treatment Automation and Radiotherapy Selection for Breast Cancer) for breast cancer RT planning. This system integrates two key components: the DMF, described in Gallego et al.,^11^ to determine whether 3D‐CRT or IMRT is the optimal technique for each patient, and a fully automated treatment planning (ATP) system to generate the corresponding treatment plan.

This study is the first to propose an end‐to‐end framework that not only automates treatment planning^21^, ^22^ but also integrates AI‐driven decision‐making and personalized selection of the RT technique. While Gallego et al.^11^ demonstrated the feasibility of AI‐based decision‐making for RT technique selection, their work did not address the subsequent planning process. In contrast, this study introduces the novel contribution of fully automating both steps—technique selection and treatment planning—within a single framework. TARS‐B ensures that the most appropriate technique is chosen for each patient and then generates a high‐quality, clinically deliverable plan with minimal planner interaction (although radiation oncologists and medical physicists remain responsible for the independent verification, evaluation, and approval of each automated plan before clinical use). To achieve its goals, TARS‐B incorporates FiF for 3D‐CRT and an AI‐guided optimization strategy for IMRT. In line with the ASTRO *Choosing Wisely* recommendations,^5^ this approach supports more efficient resource utilization while maintaining high standards of care, ultimately enhancing both consistency and efficiency in radiotherapy planning.

## 2 METHODS

The methodology used in this work was designed and conducted in accordance with the RATING guidelines for treatment planning studies,^23^ as described below.

### 2.1 Patient data

This study utilized computed tomography (CT) imaging data from breast cancer patients between 2021 and 2023 acquired in our center. Three separate cohorts of consecutive patients were selected: 30 consecutive patients treated with 3D‐CRT and 30 consecutive patients treated with IMRT. Additionally, an extra subset of 20 IMRT patients was selected to fine‐tune the parameters of the automated IMRT planning system. The groups were selected to ensure a balanced representation of anatomical variability and dosimetric complexity, which reflects the typical patient population treated in our clinic.

All patients had the same dose prescription: 40.05 Gy in 15 fractions, and only cases involving the breast without boost or nodal areas were considered. This choice was made to reduce complexity and specifically test TARS‐B framework under standard whole‐breast treatment conditions, while future versions will extend the system to include nodal irradiation. All CT images were acquired using a Brilliance Big Bore CT scanner (Philips, Andover, MA) with a resolution of 512 x 512 pixels and a 3mm slice thickness. All patients were immobilized using the Posirest (CIVCO Radiotherapy, Orange City, Iowa, USA) in a supine position with arm elevation, following standard clinical practice.

All clinical treatment plans were created using the eclipse treatment planning system (Varian Medical Systems, Palo Alto, CA), version 15.1, with the anisotropic analytical algorithm for dose calculation and a dose grid resolution of 2.5 mm. Plans were delivered on both a Clinac 2100 CD and a TrueBeam, both equipped with identical Millennium HD 120 MLCs. For consistency, all TARS‐B automated plans were generated for the TrueBeam, which represents the primary treatment platform in our institution due to its broader availability. This does not imply that the framework is restricted to a specific machine: as long as the TPS supports FiF for 3D‐CRT and IMRT optimization with consistent beam modeling and MLC configuration, TARS‐B can be applied on different linear accelerators.

Target volumes (PTV) and organs‐at‐risk (OARs) were manually delineated following the ESTRO consensus guidelines on target volume delineation.^24^ The PTV was generated by applying a 5mm isotropic expansion to the clinical target volume (CTV), while maintaining a 5mm margin from the skin. OARs delineated included the heart, ipsilateral lung, contralateral lung, and contralateral breast. Delineation was performed by experienced radiation therapy technologists under the supervision of radiation oncologists (ROs), and a peer review process was implemented to ensure consistency between practitioners.

Local ethical committee approval (REDACTED) was granted for this study, and all personal data (i.e., Dicom tags) were anonymized using the Dicompyler anonymization tool.^25^

### 2.2 TARS‐B: Automated treatment framework

**FIGURE 1:**
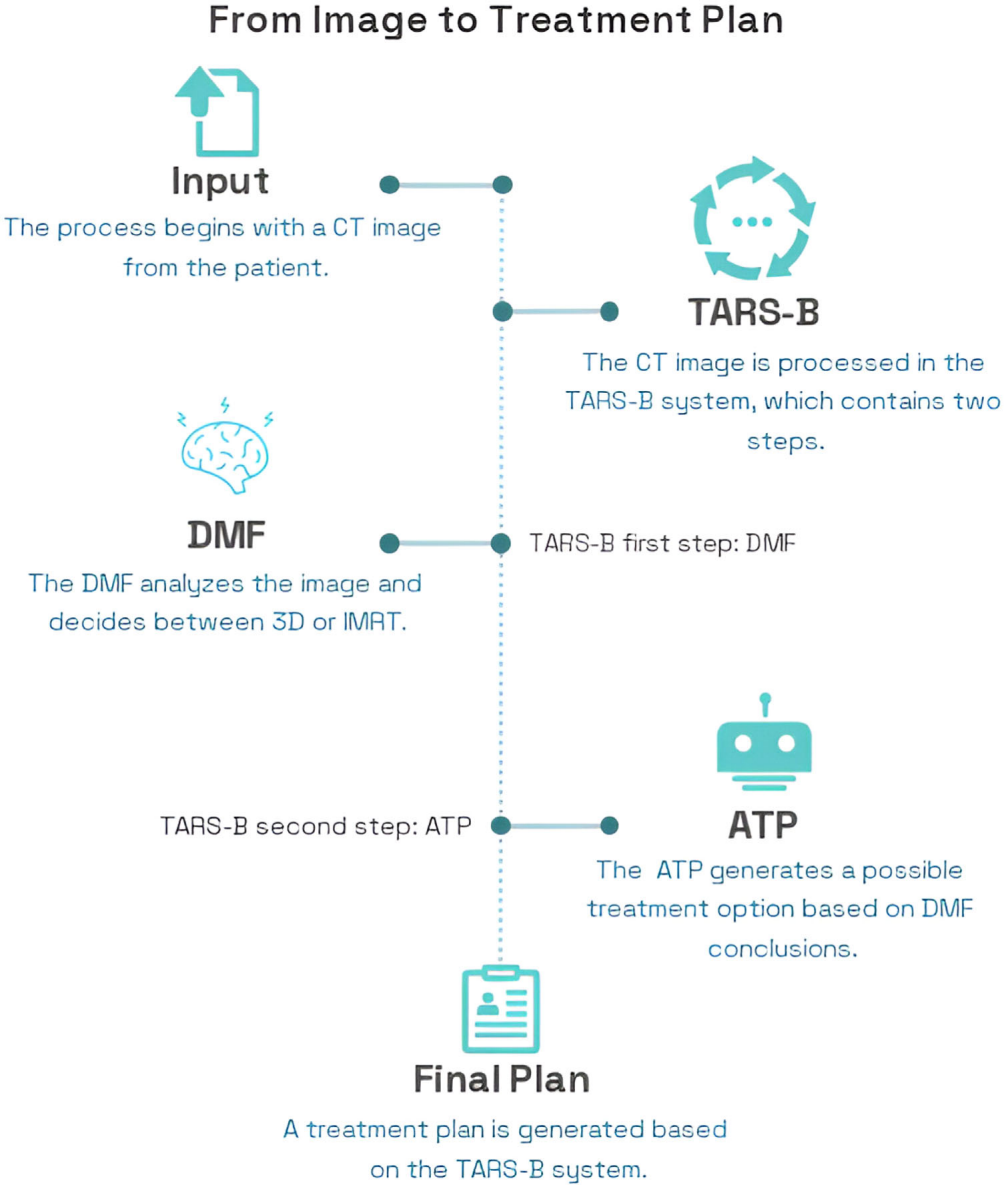
Workflow of the proposed TARS‐B system illustrating the complete AI‐driven pipeline for automated breast radiotherapy planning. The framework integrates the DMF for technique selection (3D‐CRT vs. IMRT) with the ATP module for plan generation, optimization, and evaluation. ATP, automated treatment planning; DMF, decision‐making module; IMRT, intensity‐modulated radiotherapy; TARS‐B, treatment automation and radiotherapy selection for breast cancer.

The TARS‐B developed in this study consists of two primary components: (1) a deep learning‐based DMF^11^ responsible for selecting the optimal RT technique (3D‐CRT or IMRT) for each patient, and (2) a fully ATP workflow that generate complete treatment plans based on the selected technique (Figure 1). The TARS‐B is integrated within the Eclipse TPS using scripting capabilities to ensure seamless automation of the entire planning process, minimizing the need for manual intervention.

#### 2.2.1 DMF: Technique selection: 3D‐CRT versus IMRT

The selection between 3D‐CRT and IMRT was automated using the DMF described in Gallego et al.^11^ Instead of relying on specific anatomical features, the model, based on a U‐net architecture,^26^ directly uses the patient's CT scan along with the delineated structures to predict the dose distributions for both 3D‐CRT and IMRT. Based on this information and the applied clinical constraints, the model determines which technique would be most suitable, focusing on the heart and ipsilateral lung since these are the clinically critical organs‐at‐risk in whole‐breast RT. Contralateral organs were not used in this version, but their integration will be considered in future developments.

The constraints used for this analysis were the same as those routinely applied in clinical practice, as detailed in Table 1. IMRT was selected in cases where these constraints could not be achieved with 3D‐CRT.

**TABLE 1:** Dosimetric constraints for treatment planning.

	V95%>95%
PTV	V105%<7%
	V110%=0%
	$D_{mean}<3Gy$(Left breast)
Heart	$D_{mean}<1.6Gy$ (Right breast)
	V13Gy<10%
Ipsilateral lung	$D_{mean}<10Gy$
	V18Gy<18%–20%
Contralateral breast	$D_{mean}<1.5Gy$
	V5Gy<2%

Abbreviation: PTV, planning target volume.

#### 2.2.2 Automated treatment planning

For patients selected for 3D‐CRT, the ATP system implemented a FiF strategy to improve dose homogeneity and control hotspots within the target. Beam geometry and segment weights were iteratively optimized through scripting to meet predefined coverage and heterogeneity constraints, while minimizing manual intervention.

For IMRT, ATP used a hybrid approach that combined predefined beam arrangements with neural network–based dose predictions to generate patient‐specific optimization constraints. These constraints guided the treatment planning system to achieve adequate target coverage while respecting OAR sparing.

**FIGURE 2:**
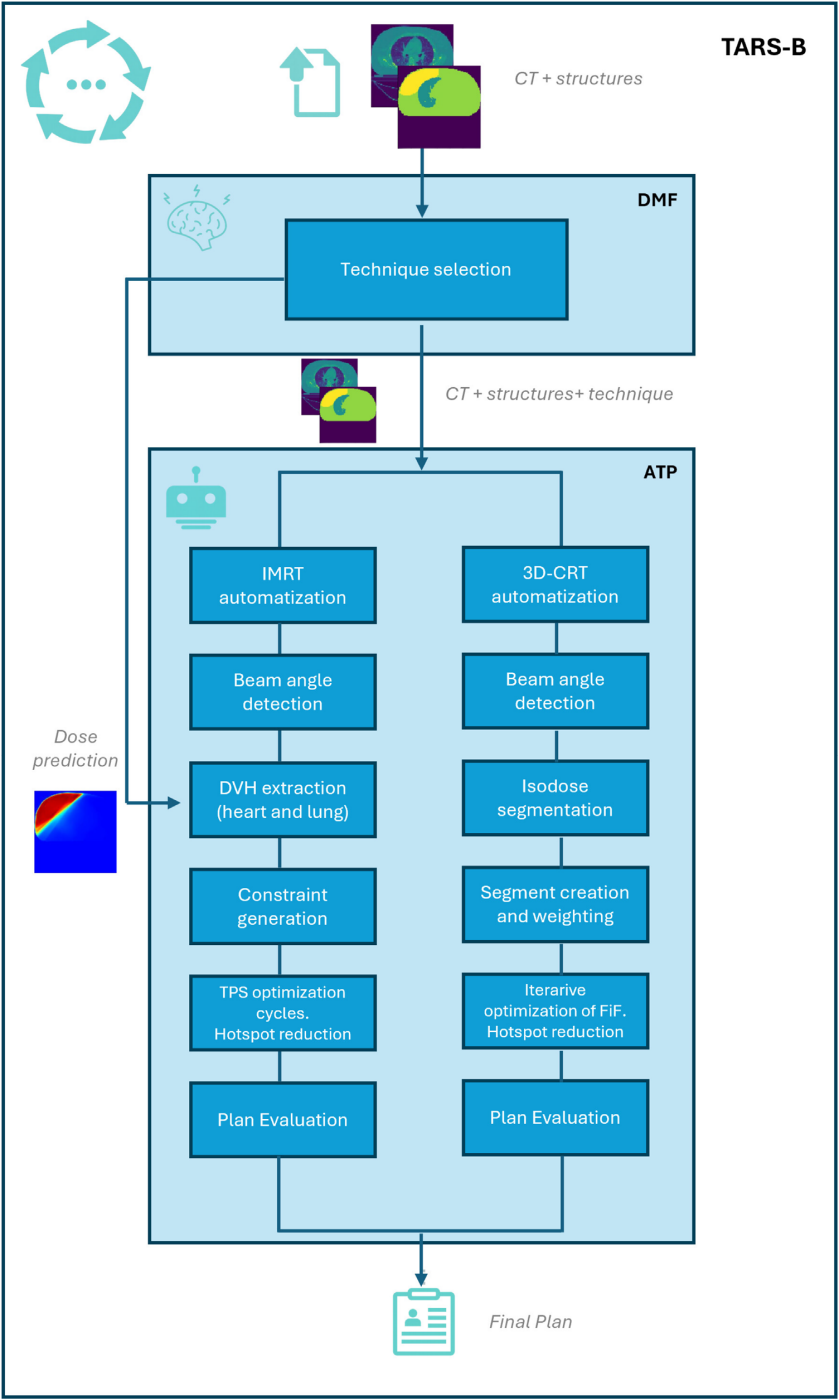
Workflow of the ATP system integrated within TARS‐B. The framework comprises two automated planning branches: the 3D‐CRT branch (right), which employs a FiF optimization to iteratively refine dose homogeneity and control hotspots, and the IMRT branch (left), which uses a neural network–based dose prediction model to generate patient‐specific optimization constraints for the TPS. ATP, automated treatment planning; FiF, field‐in‐field; IMRT, intensity‐modulated radiotherapy; TARS‐B, treatment automation and radiotherapy selection for breast cancer; TPS, treatment planning system.

Both FiF and IMRT processes were fully scripted within the Eclipse TPS, enabling reproducible plan generation with minimal planner interaction. The overall ATP workflow, summarizing both planning branches and their integration within the TARS‐B system, is illustrated in Figure 2. Full technical details of the scripting implementation and optimization stages are provided in the Appendix.

### 2.3 Dosimetric evaluation and plan comparison

To comprehensively evaluate the performance of the TARS‐B, two distinct experiments were designed. The first experiment A aims to assess whether the automated system can replicate the quality of manually generated clinical plans, ensuring its ability to meet current clinical standards (real‐world reproducibility). The second experiment B focuses on studying the impact of the full implementation of TARS‐B, evaluating whether the combination of automated technique selection and automated planning can provide dosimetric improvements compared to manual clinical plans (real‐world clinical performance).

In Experiment A, the DMF was not applied. Instead, we directly compared the automated plans generated by the ATP with the manual clinical plans previously created using either 3D‐CRT or IMRT. For each patient, the same treatment modality originally chosen in the clinic was used for both the manual and automated plans, allowing a direct comparison. All plans were evaluated based on dosimetric metrics, including PTV coverage and organs‐at‐risk constraints for the heart, ipsilateral lung, and contralateral breast, as defined in Table 1. Additionally, the number of monitor units (MU) was analyzed to assess whether the automated plans introduced increased complexity in treatment delivery. Since dosimetric parameters did not follow a normal distribution (Shapiro–Wilk test, p<0.05), the Wilcoxon signed‐rank test was used to assess statistical differences between automated and clinical plans. This non‐parametric test avoids assumptions of normality and provides a robust patient‐wise paired comparison. In addition, patient‐wise difference plots were employed to visualize the level of agreement between both planning approaches.

Additionally, all 60 automated plans were independently reviewed by a medical physicist (MP) and a RO to verify adherence to clinical standards and assess their acceptability for clinical use, without knowledge of prior clinical plans.

In experiment B, the full TARS‐B was applied. The neural network determined the optimal technique for each patient, followed by automated plan generation using the selected technique. This approach was evaluated to determine if combining automated technique selection and planning could result in dosimetric improvements over the manual approach. Key parameters, including target coverage, the constraints outlined in Table 1 for the heart, ipsilateral lung, and contralateral breast, as well as low‐dose bath (volume receiving 5 Gy) and intermediate‐dose bath (volume receiving between 5 and 20 Gy), were analyzed. Wilcoxon signed‐rank test and patient‐wise difference plots were used to identify and visualize significant differences between automated and manual plans.

In both comparisons, the time required for manual plan generation was extracted from the ARIA record‐and‐verify system (Varian Medical Systems, Palo Alto, CA), measuring the duration from the creation of the plan to its designation as the optimal plan. This was compared to the recorded times for the automatically generated plans. No plan was normalized to a specific value, ensuring that the results reflect the natural variability in clinical practice.

To account for multiple statistical comparisons, we applied a Bonferroni correction with α=0.05 and n=10, resulting in an adjusted significance threshold of p<0.005. Population values are presented with a confidence interval of one standard deviation.

## 3 RESULTS

### 3.1 Experiment A: Automated planning versus clinical planning

**FIGURE 3:**
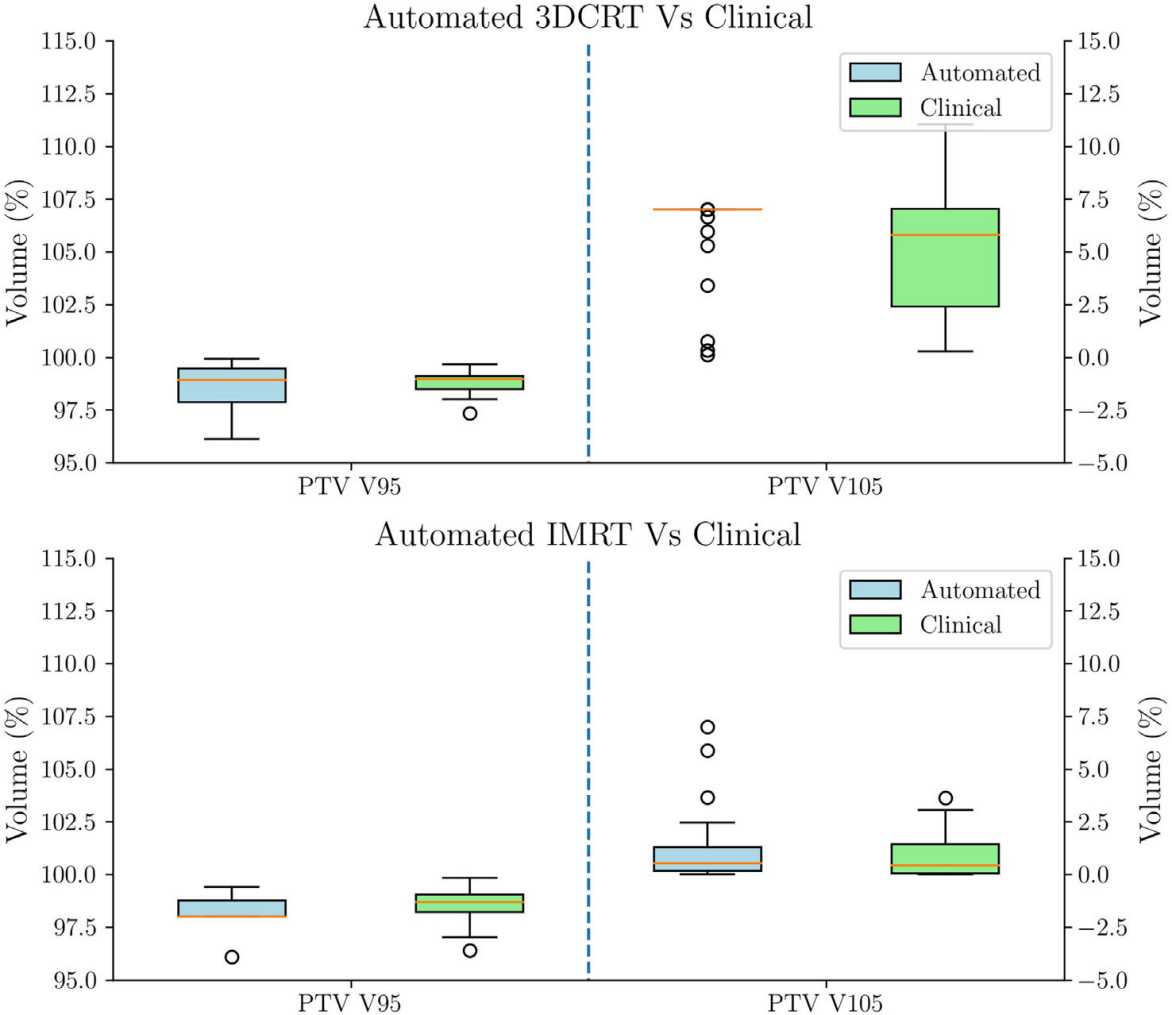
Boxplots showing PTV coverage (V95%) and hotspots (V105%) between automated and clinical plans for 3D‐CRT (top) and IMRT (bottom) patients in experiment A. Automated plans reproduced the quality of manual plans, with no significant differences observed, supporting the reliability of TARS‐B for maintaining target coverage and hotspot control.IMRT, intensity‐modulated radiotherapy; PTV, planning target volume; TARS‐B, treatment automation and radiotherapy selection for breast cancer.

**FIGURE 4:**
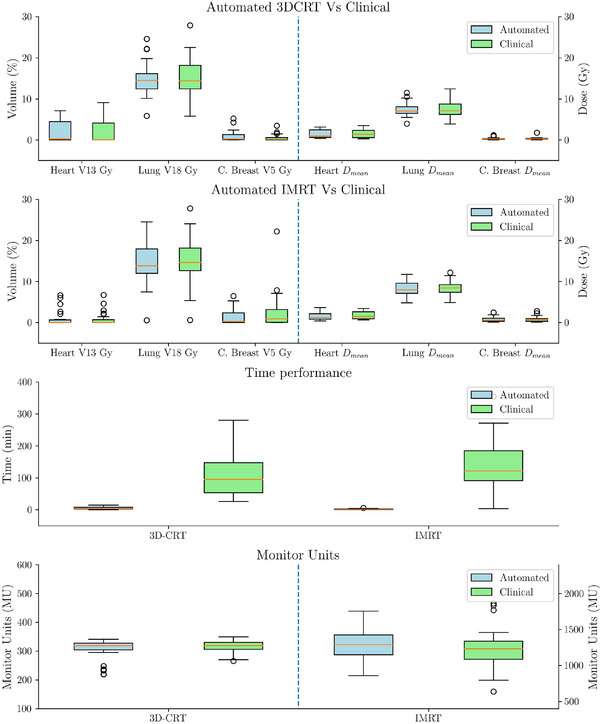
Comparison of automated and clinical plans in experiment A for 3D‐CRT and IMRT patients. The top panel shows the differences in OAR constraints for 3D‐CRT cases, while the second panel presents the same comparison for IMRT cases. The third panel compares planning time, highlighting a dramatic reduction with automation, and the bottom panel displays the differences in monitor units (MU). These results demonstrate that automation preserves dosimetric quality while substantially reducing planning workload without increasing delivery complexity. IMRT, intensity‐modulated radiotherapy; OAR, organs‐at‐risk.

**FIGURE 5:**
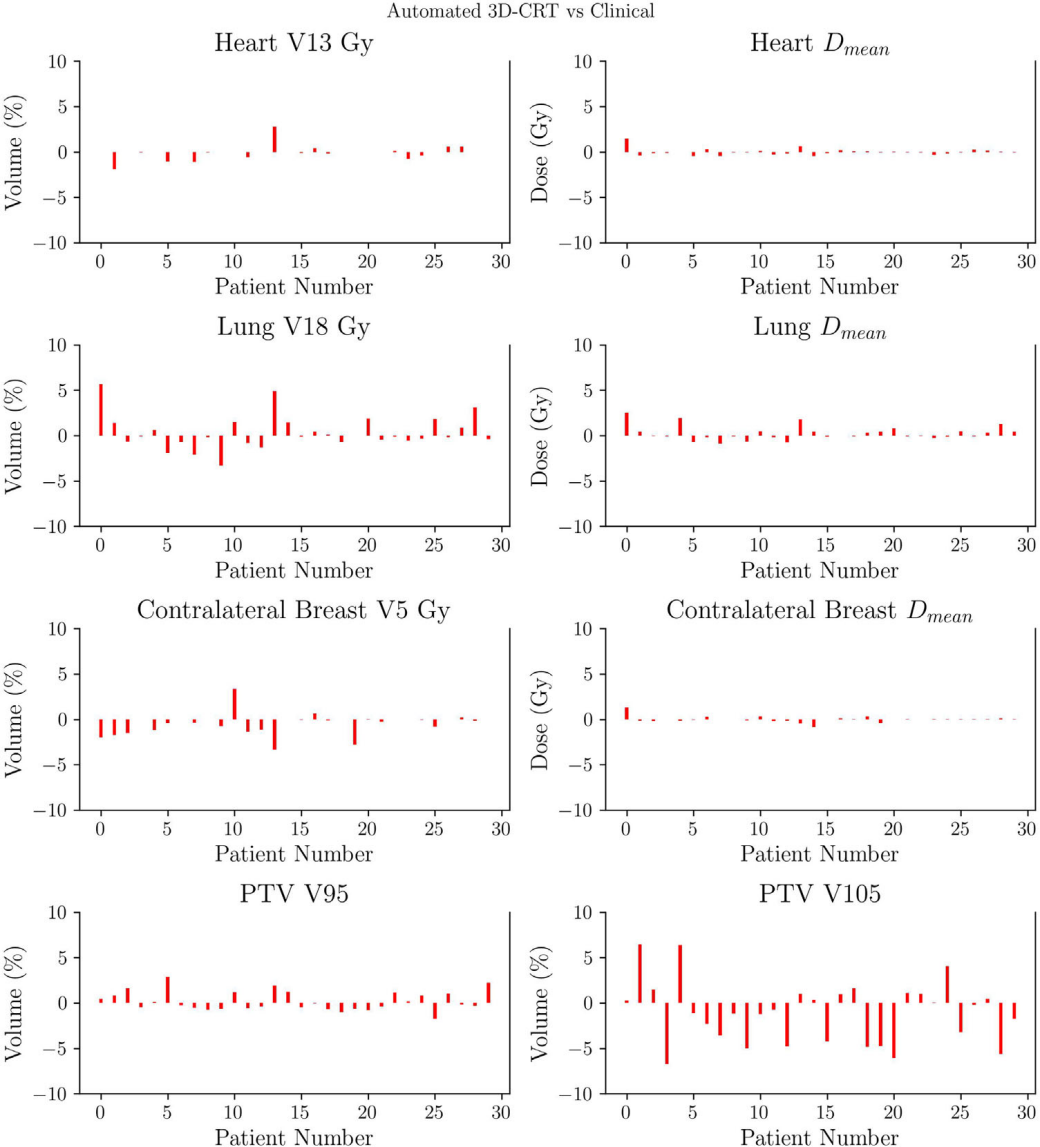
Patient‐wise difference plot showing differences between automated and clinical plans for all dosimetric metrics (OARs and PTV) for the 30 3D‐CRT patients in experiment A. Differences were minimal, indicating that automated planning achieved consistency with clinical plans on a per‐patient basis, reducing variability across the cohort. PTV, planning target volume; OAR, organs‐at‐risk.

**FIGURE 6:**
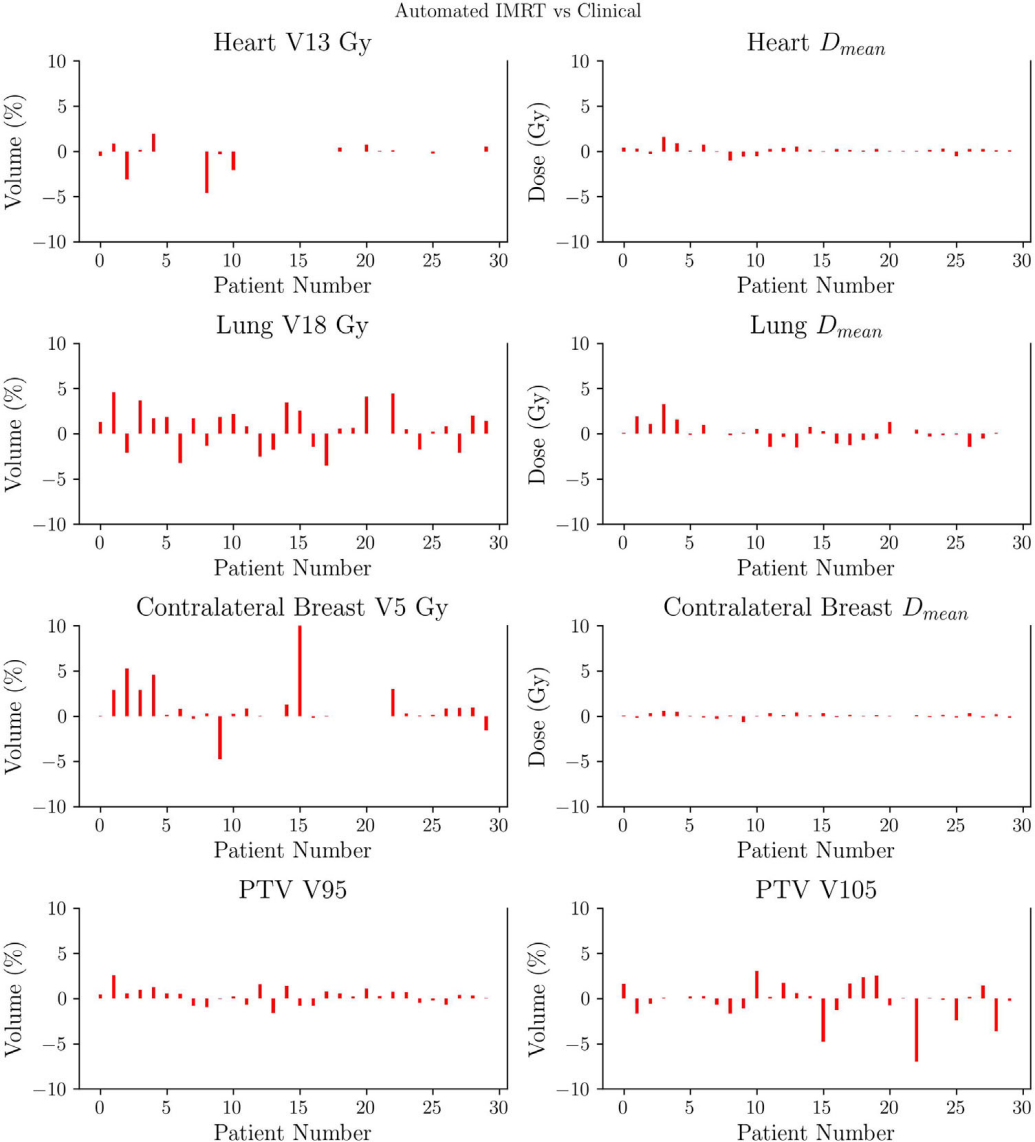
Patient‐wise difference plot showing differences between automated and clinical plans for all dosimetric metrics (OARs and PTV) for the 30 IMRT patients in experiment A. The narrow distribution of differences confirms that automated IMRT plans achieved parity with clinical plans across patients, supporting their acceptability for clinical use. IMRT, intensity‐modulated radiotherapy; PTV, planning target volume; OAR, organs‐at‐risk

**FIGURE 7:**
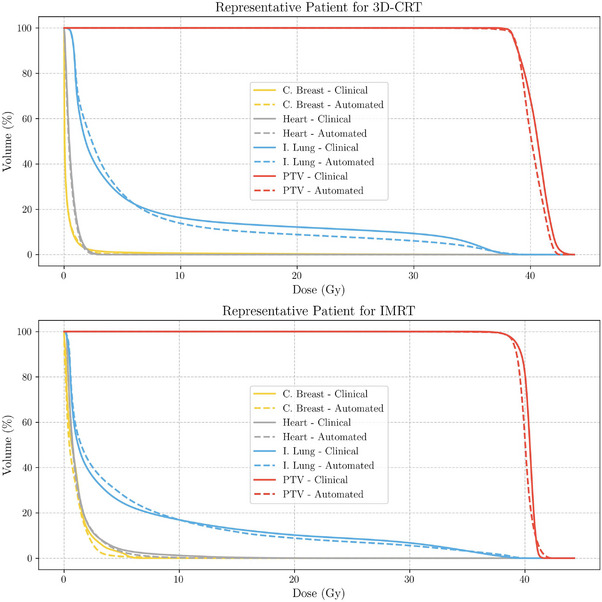
Representative DVH curves for a patient treated with 3D‐CRT (top) and IMRT (bottom). Solid lines correspond to clinical plans, while dashed lines represent automated plans. The close overlap illustrates the dosimetric similarity between automated and manual plans, confirming that automation can reliably reproduce clinical planning outcomes. DVH, dose‐volume histograms; IMRT, intensity‐modulated radiotherapy.

Automated plans were generated for 30 patients treated with 3D‐CRT and 30 with IMRT, replicating the treatment modality originally chosen for treatment. Figures 3 and 4 present boxplot representations of the constraints detailed in Table 1. Such results do not show statistically significant differences between automated and manual plans for any of the evaluated dosimetric parameters (p>0.005). Patient‐wise difference plots in Figures 5 and 6 further illustrate the agreement between automated and manual plans, confirming minimal deviations across key metrics, including MU. Representative DVH curves for a 3D‐CRT and an IMRT patient are depicted in Figure 7.

Automated planning significantly reduced average planning times observed in the clinic for both techniques, with IMRT planning dropping from 157.4±116.2 minutes to 2.0±1.3 min, and 3D‐CRT planning decreasing from 112±70 minutes to 5±4 min (p<0.005).

All automated plans were reviewed and accepted by a MP and a RO without further adjustments, demonstrating the reliability of the TARS‐B in producing clinically viable plans.

### 3.2 Experiment B: Decision‐making framework implementation

In the second comparison, the TARS‐B was applied to determine the optimal technique and generate treatment plans for each patient. For the 30 patients initially treated with 3D‐CRT, the TARS‐B confirmed that 3D‐CRT was the optimal technique in all cases. Among the 30 patients initially treated with IMRT, the TARS‐B identified 15 cases suitable for 3D‐CRT. Automated plan generation for 3D‐CRT was successfully completed for 14 patients. One plan did not meet dosimetric criteria due to the use of a bolus, representing a geometric outlier not accounted for in the model training.

**FIGURE 8:**
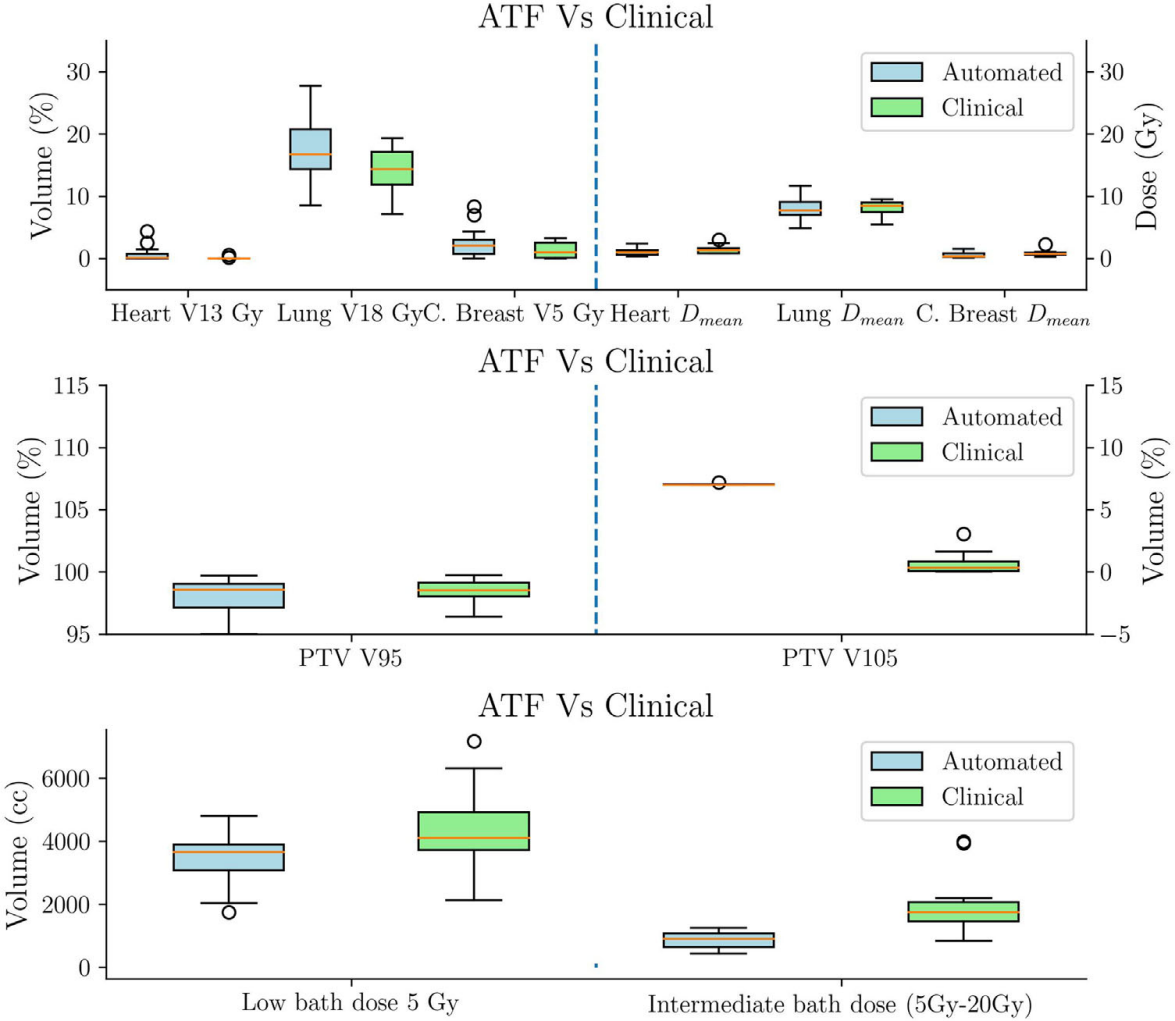
Differences in OAR constraints (Table 1), low‐dose bath (volume receiving 5 Gy), and intermediate‐dose bath (volume receiving 5–20 Gy) for 14 IMRT patients re‐planned as 3D‐CRT using the decision‐making framework (Experiment B). The reduction in intermediate‐dose bath observed in automated re‐planning may translate into a clinically relevant reduction in pulmonary and cardiac toxicity. IMRT, intensity‐modulated radiotherapy; OAR, organs‐at‐risk.

**FIGURE 9:**
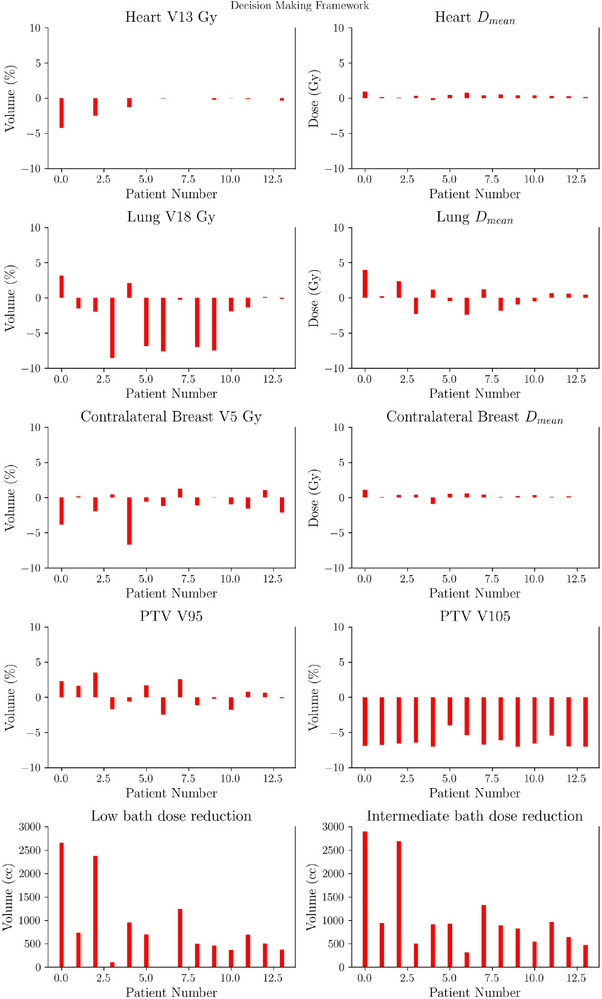
Patient‐wise difference plot showing differences between automated and clinical plans for 14 IMRT patients re‐planned as 3D‐CRT using the decision‐making framework (Experiment B). The results highlight that the DMF can safely redirect selected IMRT cases to 3D‐CRT without compromising target coverage or OAR protection, thereby favoring more efficient treatment in standard scenarios.DMF, decision‐making module; IMRT, intensity‐modulated radiotherapy; OAR, organs‐at‐risk.

Figure 8 summarizes the dosimetric metrics for the 14 re‐planned patients. Patient‐wise difference plots in Figure 9 illustrate the agreement between automated and clinical plans. No statistically significant differences were observed for heart and lung constraints, or for target coverage, except for V105% of the PTV, which was higher in automated plans (p<0.005).

The increase in V105% remains within our institutional threshold (<7%) and represents a trade‐off for improved target coverage. This is acceptable but may be adjusted depending on institutional policies.

Notably, automated plans achieved significant reductions in low and intermediate‐dose bath volumes (p<0.005). The reduction of up to 3000 ${\mathrm{cm}}^{3}$ in the intermediate‐dose bath is clinically significant, as it has been associated with a decreased risk of pneumonitis and secondary malignancies.

## 4 DISCUSSION

The results of this study highlight the efficacy of the TARS‐B in automating breast radiotherapy planning using a single CT image and the delineated structures as input, while maintaining high‐quality clinically relevant dosimetric performance. The TARS‐B consistently produced treatment plans that matched the quality of manual clinical plans, achieving significant reductions in planning time while maintaining dosimetric performance across key metrics (e.g., 111.9 to 5.1 min in 3D‐CRT, and from 157.4 to 2.0 min in IMRT).

Automated system against manually generated clinical plans were investigated. No significant differences (p>0.005) were observed across key dosimetric parameters, including PTV coverage and OAR constraints. This outcome underscores the ability of the TARS‐B to replicate the quality of manual plans, validating the robustness and reliability of the automated process. The close agreement between automated and manual plans, as illustrated in Figures 4 and 3, suggests that automation can streamline clinical workflows without compromising treatment quality.

An important aspect evaluated in this comparison was the analysis of MU for automated and clinical plans (Figure 4). Despite the automation process, no significant differences were observed in MU values, suggesting that the implementation of the TARS‐B does not introduce additional complexity.

In the experiment B, which involved re‐planning patients originally treated with IMRT using the TARS‐B, reductions of up to 2800 ${\mathrm{cm}}^{3}$ in low‐dose bath and 3000 ${\mathrm{cm}}^{3}$ in intermediate‐dose bath were observed, representing statistically significant improvements (p<0.005) in both cases. Such reductions in bath dose are clinically relevant, as prior studies have linked high bath doses to increased risks of pneumonitis and secondary malignancies.^27^ Importantly, despite the reduction in bath dose, no statistically significant differences in key OAR metrics were observed, supporting the hypothesis that automation can improve tissue sparing without compromising target coverage or OAR metrics.

A notable observation in the re‐planned cases was the increase in PTV V105% for 3D‐CRT plans compared to IMRT plans. The automated framework consistently adjusted V105% to approximately 7% to maximize PTV coverage, aligning with our institutional guidelines and constraints. While this threshold is acceptable within our clinical criteria, it is important to note that this parameter can be adjusted within the TARS‐B framework to accommodate different institutional preferences or specific patient requirements. However, reducing this threshold would likely result in an increase in the number of segments.

The efficiency gains provided by the TARS‐B were another key finding. Planning times for 3D‐CRT were reduced from 111.9±68.4 to 5.1±4.0 min, while IMRT planning times decreased from 157.4±116.2 to 2.0±1.3 min. Manual planning times were extracted from the ARIA record‐and‐verify system, which may not fully capture the total time required for manual plan creation. However, it serves as a reliable estimator of the time needed in a real clinical environment. These results are consistent with previous studies that demonstrated substantial reductions in manual workload and planning variability through automated frameworks.^21^, ^22^ Such improvements have a direct impact on resource allocation, allowing planners to focus on complex cases requiring manual intervention.

Similar findings have been reported in large studies of automated planning. Hazell et al.^22^ showed that head‐and‐neck autoplans were clinically acceptable in all 26 cases and rated at least as high as clinical plans in 94% of blinded evaluations, while Hansen et al.^21^ reported that automatic plans were selected for treatment in 29/30 consecutive patients, achieving more homogeneous target doses, significant OAR sparing, and approximately halving active planning time. Compared with these results, TARS‐B achieved equivalent plan quality in breast radiotherapy with even larger relative efficiency gains, and uniquely integrates AI‐based modality selection to further optimize the choice between 3D‐CRT and IMRT.

Previous studies have investigated the use of deep learning for dose prediction. Nguyen et al.^7^ and Chen et al.^8^ demonstrated that convolutional neural networks can accurately predict 3D dose distributions and may inform plan optimization, and Bakx et al.^20^ applied similar approaches to breast cancer. However, these works were limited to prediction and did not generate clinically deliverable plans. The present study advances the field by integrating deep learning into an end‐to‐end automated workflow that not only selects the treatment technique but also produces complete treatment plans ready for clinical evaluation. Although efficiency gains were achieved, manual quality assurance by radiation oncologists and medical physicists remains essential. Adaptation to different TPS versions and validation in multicenter settings are required before routine clinical deployment.

Despite these positive outcomes, certain limitations are observed in this work. While the neural network focuses on predicting dose distributions for the heart and ipsilateral lung—excluding contralateral structures such as the opposite lung and breast—this choice has minimal impact on technique selection, as the limiting organs in breast radiotherapy are typically the heart and ipsilateral lung. Nevertheless, including these additional OARs in future versions could enhance the comprehensiveness of the dose predictions.

Additionally, although the neural network was trained on patients both with and without lymph node involvement, in this study we deliberately limited the analysis to the breast PTV. This decision was made to reduce complexity and clearly demonstrate the effectiveness of the TARS‐B framework in guiding treatment technique selection and automated planning. Future work will explore the integration of nodal volumes to broaden the clinical applicability of the model, at which point the inclusion of VMAT will also be investigated given its reported advantages in complex locoregional breast cancer cases.

Furthermore, the TARS‐B has not yet undergone validation in external institutions. Multi‐center validation will help ensure consistent performance and adaptability to diverse workflows. Future research should focus on expanding validation efforts and refining the framework to address more complex cases, including patients with unique anatomical variations or bolus applications.

Beyond these technical aspects, broader considerations must be acknowledged. Lahmi et al.^28^ emphasized ethical issues such as transparency and accountability, while Hallows et al.^29^ and others^30^, ^31^ highlighted the need for explicit human oversight and robust QA protocols before routine adoption. These perspectives underline that frameworks such as TARS‐B should be regarded as workflow optimization and decision‐support tools rather than substitutes for clinical expertise.

Overall, the proposed TARS‐B demonstrates strong potential to improve breast radiotherapy planning by automating and standardizing key components of the current clinical workflow. Addressing the identified limitations will further enhance its clinical utility and ensure that the benefits observed in this study can be generalized to a broader patient population.

## 5 CONCLUSION

This study demonstrates the feasibility and clinical value of integrating automated technique selection with automated treatment planning in breast radiotherapy. The proposed TARS‐B framework successfully reproduces the quality of manual plans, minimizes intermediate bath dose, and substantially reduces planning time. By streamlining the workflow and minimizing human intervention, TARS‐B has the potential to alleviate clinician workload and improve the consistency of treatment planning. Importantly, TARS‐B may also contribute to the clinical adoption of the *Choosing Wisely* principles by limiting the use of IMRT to anatomically complex cases and favoring 3D‐CRT in standard situations, thereby achieving efficiency gains without compromising plan quality.

## AUTHOR CONTRIBUTIONS

P.G., E.A., and J.P‐A. designed the study and developed the automated planning framework. P.G. and A.M. implemented the scripting system and performed the experiments. E.A. and J.P.‐A. supervised clinical integration. J.C.J., A. Mera and the Radiation Oncology Department contributed with clinical expertise. S.B. and O.D. supported the AI model development and integration. All authors contributed to manuscript writing and approved the final version.

## CONFLICT OF INTEREST STATEMENT

The authors declare no known competing financial interests or personal relationships that could have appeared to influence the work reported in this paper.

## APPENDIX A ATP 3D‐CRT: Field‐in‐field automated planning

For patients treated with 3D‐CRT, the TARS‐B employed a FiF technique to enhance dose homogeneity and reduce hotspots within the PTV. The FiF method was implemented through an automated scripting solution within the Eclipse TPS. The script, coded in C#, accept as input two constraints: a coverage criterion (e.g., V95% > 98%) and a heterogeneity criterion (e.g., V105% < 7%, Dmax < 110%). The process was divided into several stages to ensure that these dosimetric constraints were met with minimal manual intervention:

### A.1 FiF stage I

In this first stage, the system can operate in fully automatic mode, where it proposes two tangential beams—internal tangential and external tangential—by automatically selecting their angles. The angles are determined by identifying the most lateral and most inferior pixels of the PTV and drawing a tangent line. Alternatively, the user can choose to start the optimization process with a predefined beam arrangement instead of the automatically generated one. Once the angles are selected, dose calculation is performed, and the plan is normalized to achieve the desired PTV coverage.

Following normalization, isodose structures are generated at 5% intervals starting from 105%, and for each isodose level, a pair of segments—one internal and one external—is created. These segments share the same geometric characteristics as the tangential beams but are specifically shaped to cover their corresponding isodose region. The MLCs are adjusted accordingly, and the segment weights are systematically explored to maximize PTV coverage while minimizing hotspots (heterogenity criterion). The number of segments generated can be customized; in our configuration, the system was set to generate a maximum of 10 segments, corresponding to five isodose levels from 105% to 130% in 5% intervals. This range was chosen as a clinically acceptable compromise between plan quality and computational efficiency. If the coverage and heterogeneity constraints are not met by the end of this process, TARS‐B advances to the next phase.

### A.2 FiF stage II

In this stage, the system addresses cold spots—areas within the PTV that receive less than the prescribed dose. To correct these underdosed regions, a structure is generated to delineate the cold spot, and an additional segment is created specifically to improve dose coverage in that area. The weight of this segment is incrementally adjusted to enhance PTV coverage while avoiding the introduction of new hotspots. If the coverage constraints are still not met, the system proceeds to optimize beam angles by varying them in 10 increments. This angular adjustment is repeated up to four times. Beyond this limit, the resulting beam directions would be too direct, leading to suboptimal dose distributions and potentially higher exposure to surrounding healthy tissues—conditions that are not acceptable in clinical practice. If, after all iterations, the coverage criteria are still unmet, the system assumes that a fully optimal plan cannot be achieved automatically; however, the best available plan is still generated for planner review and potential manual adjustment.

## APPENDIX B ATP IMRT: AI‐guided optimization

The automated IMRT planning system, like the FiF technique, is implemented within the scripting environment of the Eclipse TPS. For patients selected for IMRT, the automated planning process begins by selecting tangential beams following the same methodology as in the FiF technique. In addition to these tangential beams, four more beams are added—two on the internal side and two on the external side—with increments of 10 from the initial tangential angles. As for the FiF scenario, the user can choose to start the process with a predefined beam arrangement instead of the automatically generated one.

Once the beam angles are selected, the neural network^11^ prediction of the three‐dimensional dose distribution is used. From this prediction, dose‐volume histograms (DVHs) for the heart and ipsilateral lung are extracted. The automated system uses these predicted DVHs to generate 20 upper constraints, evenly distributed between 0 Gy and the maximum predicted dose for each structure. The choice of 20 constraints was determined empirically using a grid search approach, where the number of constraints was systematically varied between 10 and 30 to evaluate its impact on plan quality. The evaluation was based on the intrinsic cost function of the optimizer, which balances target coverage, dose homogeneity, and organ sparing. The results showed that increasing the number of constraints beyond 20 did not lead to a significant improvement in the optimized plans but instead introduced excessive rigidity, limiting the optimizer's ability to adjust trade‐offs effectively. However, the exact number of constraints is not critical as long as there are sufficient points to accurately reproduce the shape of the dose‐volume curve. The selected value of 20 constraints provides a good trade‐off between computational efficiency and fidelity in capturing the predicted dose distribution.

The weights of these constraints were not predefined but rather optimized using a grid search. Initially, a range of possible weights was established, and a grid search approach was used to systematically explore different combinations. The selection criterion was based on finding the set of weights that best replicated the dose distributions predicted by the neural network, while maintaining clinically acceptable trade‐offs between target coverage and organ sparing. The number of superior constraints and the final weights, detailed in Table B.1, were fine‐tuned using the separate set of 20 patients not included in the primary dataset.

**TABLE B.1:** Weights assigned to heart and lung constraints.

Constraint index	Heart weight	Lung weight
1	200	40
2	300	200
3	200	300
4	250	300
5	200	300
6	150	200
7	150	200
8	150	200
9	150	200
10	100	150
11	100	150
12	100	150
13	60	100
14	40	100
15	10	50
16	10	50
17	10	10
18	10	10
19	10	10
20	10	10

For the remaining OARs—contralateral breast, contralateral lung, and spinal cord—generalized equivalent uniform dose (gEUD) constraints with fixed values were applied. The selection of these parameters was guided in part by the recommendations reported by Fogliata et al.^32^ Specifically, a gEUD with a=2 was used for the contralateral lung to emphasize dose reduction across the entire volume, similarly to controlling the mean dose. In contrast, a gEUD with a=20 was applied to the contralateral breast and spinal cord to place stronger emphasis on limiting high‐dose regions. All three structures were constrained to 0 Gy, with assigned priorities of 120, 140, and 140, respectively. Additionally, the PTV was constrained to receive exactly 40.05 Gy, with a lower bound ensuring 100% of the volume received at least this dose (priority 350), and upper and mean dose constraints fixed at 40.05 Gy with priorities of 300 and 100, respectively.

The process begins with an initial optimization cycle until convergence is reached. Once the optimization stabilizes, TARS‐B analyzes the plan for regions receiving doses >105 % (hotspots). If hotspots are detected, TARS‐B automatically contours these areas and initiates a new optimization cycle to reduce them. This process is repeated up to a configurable number of iterations (*N* = 4 in our set‐up), or until the hotspots are sufficiently mitigated (i.e., V105% < 7%) while maintaining acceptable target coverage and organs‐at‐risk sparing.
